# Biomass accessibility analysis using electron tomography

**DOI:** 10.1186/s13068-015-0395-8

**Published:** 2015-12-25

**Authors:** Jacob D. Hinkle, Peter N. Ciesielski, Kenny Gruchalla, Kristin R. Munch, Bryon S. Donohoe

**Affiliations:** Computational Science Center, National Renewable Energy Laboratory, 15013 Denver West Parkway, Golden, CO 80401 USA; Biosciences Center, National Renewable Energy Laboratory, 15013 Denver West Parkway, Golden, CO 80401 USA

**Keywords:** Accessibility, Porosimetry, Tomography, Cellulose, Pretreatment, Biomass

## Abstract

**Background:**

Substrate accessibility to catalysts has been a dominant theme in theories of biomass deconstruction. However, current methods of quantifying accessibility do not elucidate mechanisms for increased accessibility due to changes in microstructure following pretreatment.

**Results:**

We introduce methods for characterization of surface accessibility based on fine-scale microstructure of the plant cell wall as revealed by 3D electron tomography. These methods comprise a general framework, enabling analysis of image-based cell wall architecture using a flexible model of accessibility. We analyze corn stover cell walls, both native and after undergoing dilute acid pretreatment with and without a steam explosion process, as well as AFEX pretreatment.

**Conclusion:**

Image-based measures provide useful information about how much pretreatments are able to increase biomass surface accessibility to a wide range of catalyst sizes. We find a strong dependence on probe size when measuring surface accessibility, with a substantial decrease in biomass surface accessibility to probe sizes above 5–10 nm radius compared to smaller probes.

## Background

The challenges of efficiently deconstructing lignocellulosic biomass stem largely from the complex chemical and physical interactions among the cell wall polymers [1, 2]. Substrate accessibility to catalysts has been a dominant theme in biomass deconstruction and several groups have concluded that enabling the accessibility of the biopolymers of plant cell walls remains the single most important challenge of biomass deconstruction [3–6]. Thermochemical pretreatments using acid or base chemistries represent the simplest and smallest in the range of catalysts that are used to deconstruct biomass cell walls [7]. Following pretreatment, the most commonly used catalyst system for biomass deconstruction is the small, secreted enzymes from cellulolytic fungi, such as *Trichoderma reesei* [8]. These enzymes range from 10–12 nm in their largest dimensions. Along the size and complexity continuum, the next catalytic system is the multifunctional, multidomain enzymes such as CelA from the thermophilic bacterium *Caldicellulosiruptor bescii* [9]. CelA is 15–30 nm in radius. An even larger and more complex cellulase system is found in the cellulosomes. These multi-enzyme macromolecular complexes are produced by several cellulose-degrading anaerobic bacteria including *Clostridium thermocellum* whose cellulosomes range in size from 50–70 nm radius [10, 11]. Recent studies have indicated that cellulosomes do exploit different mechanisms of interaction with cellulose substrates to affect their deconstruction [12, 13]. As the size and complexity of these catalytic systems increases, they also increase in flexibility and variability of conformation. At the extreme end of the size spectrum is the case of biomass deconstruction by whole microbes that keep their cellulose-degrading enzymes directly tethered to their own cell surface [14]. In this case, the entire system is typically on the order of 2–5 microns in size [15].

Because of the complex architecture of plant cell walls and the fact that both chemical and physical properties change during deconstruction, no one approach has become the standard for accessibility measurement. A seminal work on measuring the porosity of living plant cell walls was performed by Carpita et al. [16]. They used solute exclusion to determine the pore size of cell walls from several plant cell types to range between 3.5 and 5.2 nm. Recent work has used solute exclusion to probe the changes in porosity caused by dilute acid pretreatment [17]. Another commonly used technique is Simons’ stain, which employs two differently sized molecular stains in tandem to interrogate the range of accessibility created in pretreated materials [18]. An overview of these and more recent methods involving NMR and mercury intrusion has been reported by Meng and Ragauskas [19].

Imaging has also been used to directly visualize and measure the complex pore structure of biomass cell walls [6]. Typically field emission scanning electron microscopy (FE-SEM), atomic force microscopy (AFM), and transmission electron microscopy (TEM) are the techniques capable of sufficient spatial resolution to visualize the scale of porosity that impacts catalysts. Because cell walls are structured 3D nanomaterials, the application of 3D electron tomography is especially well suited to visualize its changing architecture.

The first example of using 3D electron tomography to analyze cell wall architecture used mildly treated pine cell walls and manual segmentation to identify and localize cellulose microfibrils and the hemicellulose/lignin matrix material surrounding individual and bundled microfibrils [20]. Subsequently, electron tomography has been used to visualize the phenomena of lignin coalescence and relocalization and the creation and distribution of new void space caused by pretreatments [6, 21]. The geometries of individual microfibrils and bundles were measured and modeled from electron tomography data [22]. In the most recent example of electron tomography of cell walls, Sarkar et al. present a comprehensive look at the impact of different cryo-immobilization techniques on the preservation of cell wall structure [23]. In that study and other work [24] that group examines the use of manual, semi-automated, and fully automated segmentation techniques in analyzing electron tomography data sets from plant cell walls and finds that semi-automated methods are able to provide similar results as manual delineation with considerably less effort.

In this work we develop novel, quantitative accessibility measures using 3D electron tomography. These methods enable quantitative comparisons of accessibility across datasets for a wide range of catalyst sizes. Applying them to biomass samples in various states of pretreatment, we find that these methods provide a nuanced picture of accessibility. Catalyst size is observed to have a dramatic impact on accessibility; catalysts whose radii are below around 10 nm enjoy greatly enhanced accessibility to biomass following pretreatment, while larger catalysts face accessibility similar to that of native biomass.

## Results and discussion

**Fig. 1:**
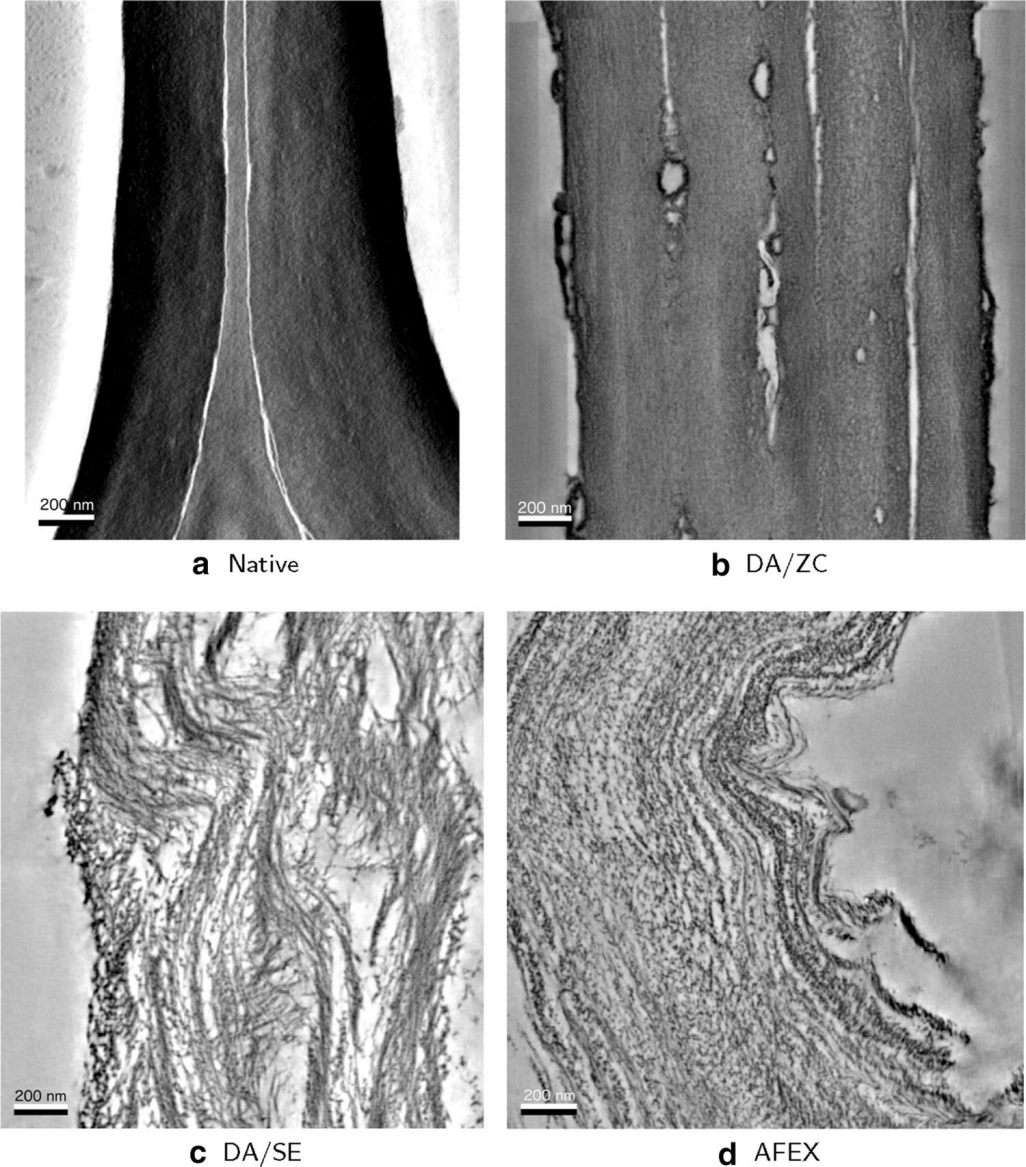
Corn stover tomography data. Four tomograms shown in 2D slices for corn stover samples in the following pretreatment conditions: **a** native, **b** dilute acid + zipper-clave, **c** dilute acid + steam explosion, **d** AFEX. *Scale bars* 200 nm

In this work, biomass accessibility is characterized quantitatively by a novel method using a 3D electron tomogram as input (see Fig. 1). In this section, we first describe this novel characterization method, and then demonstrate its effectiveness on actual tomography data.

**Fig. 2:**
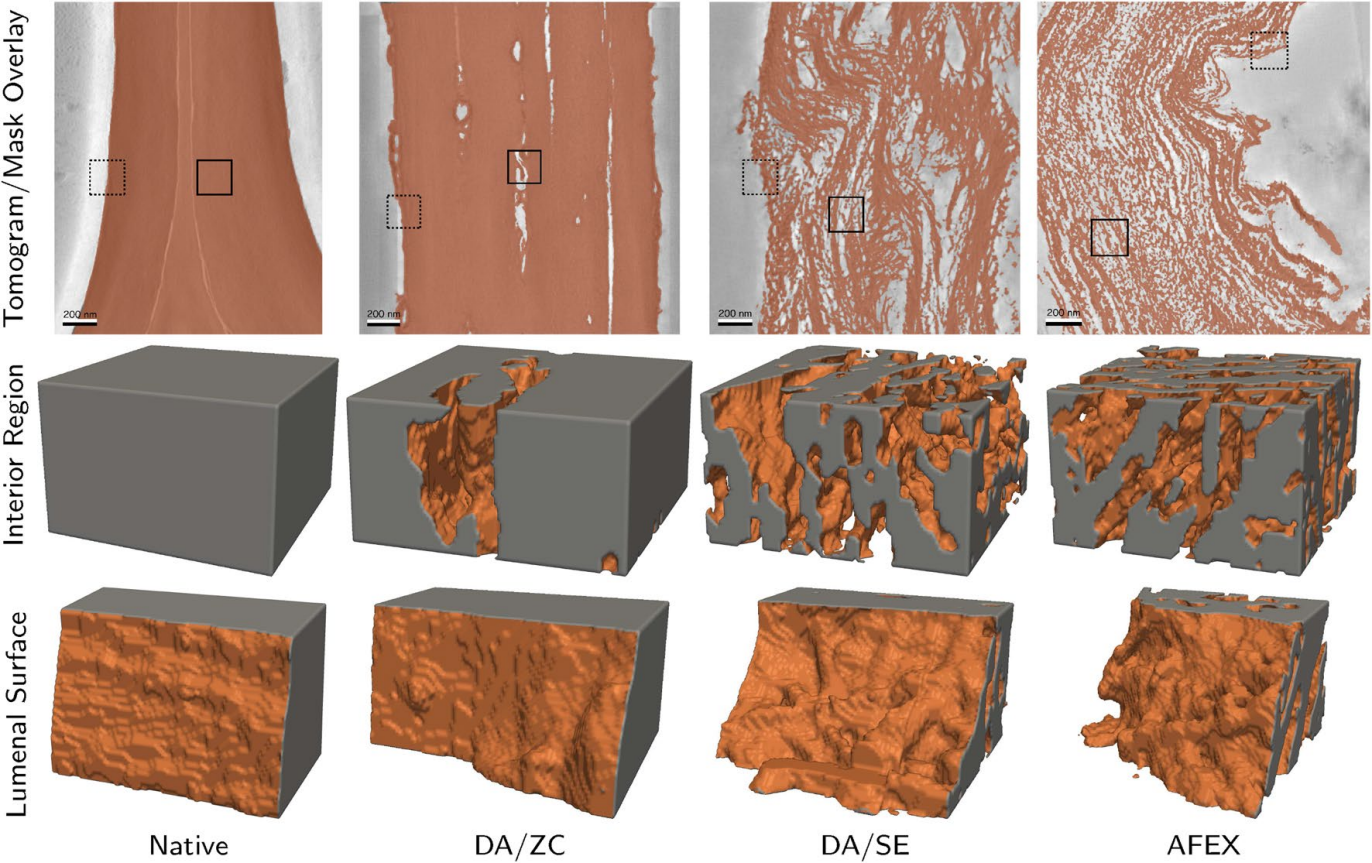
3D view of segmentations. In each panel of the* top row*, a slice of the tomogram is shown in *grayscale* with the corresponding slice of the 3D segmentation mask overlaid in *orange*. Below, 3D cutouts are shown for subregions of each of four datasets. The solid rectangular regions in the slices in the* top row* indicate the interior subregions shown in the* middle row* while the *dashed* rectangular regions indicate the luminal surface subregions shown on the* bottom row*. The datasets present different degrees of internal cavitation as well as different surface roughnesses. *Scale bars* 200 nm

Our method begins by segmenting a tomogram into biomass and void space regions using a semi-automatic segmentation method as described in “Biomass segmentation” section. The resulting segmentation provides not only a volumetric label map describing the type of each voxel (biomass or void space), but is used to compute the biomass surface, as seen in Fig. 2. As described in more detail in the remainder of this section, we compute the amount of that biomass surface which is accessible to catalysts of a given size (or smaller), and report that surface area for a wide range of catalyst sizes.

In the following discussion, $\varOmega \subset {\mathbb{R}}^{3}$ will denote the rectangular domain of a three-dimensional tomogram. ${\text{Biomass}} \subset \varOmega$ will denote the biomass region found within the tomogram, which is obtained by semi-automatic segmentation as described in “Biomass segmentation” section.

### Accessible covering radius transform

The covering radius transform (CRT) has previously been used in materials science contexts to quantitatively measure the pore size distribution using imaging data [25, 26] and to measure the thickness of solid objects such as trabecular bone [27]. In this section, we first review the Euclidean distance transform (EDT) and the CRT. Then, we present a modified version of the CRT that lends information particularly suited to quantifying catalyst access to biomass in plant cell walls, which we call the accessible covering radius transform (aCRT).

**Fig. 3:**
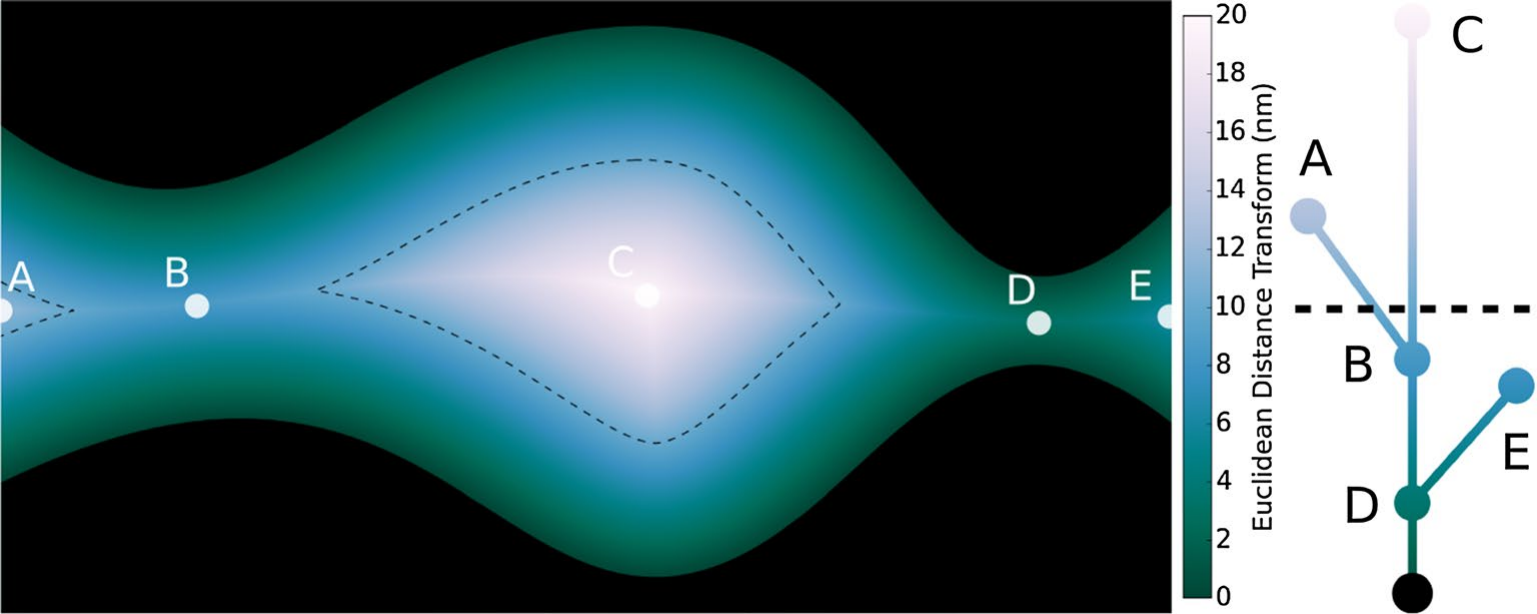
The Euclidean distance transform contour tree. The Euclidean distance transform (*left*) shows the distance from each point in the void space to the nearest point of biomass (*black*). Critical values are shown as *labeled points A–E* on the EDT figure. At *right* is the EDT contour tree. Note that *critical points* in the EDT correspond to leaves and branch points in the contour tree, and that the *vertical position* of nodes in the contour tree correspond to EDT values of those critical points (also depicted with *colors* on the contour tree). The *dashed curve* on the EDT corresponds to a particular level set of the EDT, and clearly contains two connected components. Due to the vertical ordering of the tree, those components are also visible as the intersection points of the *dashed isoline* with the contour tree

The Euclidean distance transform, EDT : *Ω* *→* *R*, assigns to each point in the image domain the distance to the nearest piece of biomass

$${\tt{EDT}}\left( x \right) = { \hbox{min} }\{ |x - y|:y \in {\text{Biomass}}\} ,$$

where the norm |*x* − *y*| indicates the Euclidean distance between the points *x* and *y*. EDT(*x*) is the largest radius of a hypothetical spherical particle that could be placed at *x* without any part of the particle overlapping biomass. The EDT has found widespread use in image analysis, and algorithms exist for computing it efficiently [28, 29]. In the left panel of Fig. 3, a hypothetical 2D pore is shown, with biomass in black and the EDT represented via color in the void space. Notice that the EDT varies continuously, being zero at the biomass surface and maximal in the interior of channels and pores. As depicted in the tree structure, the critical values of the EDT form a complex hierarchical structure called a contour tree, which will be discussed in more detail in “Computing the aEDT using the contour tree” section.

**Fig. 4:**
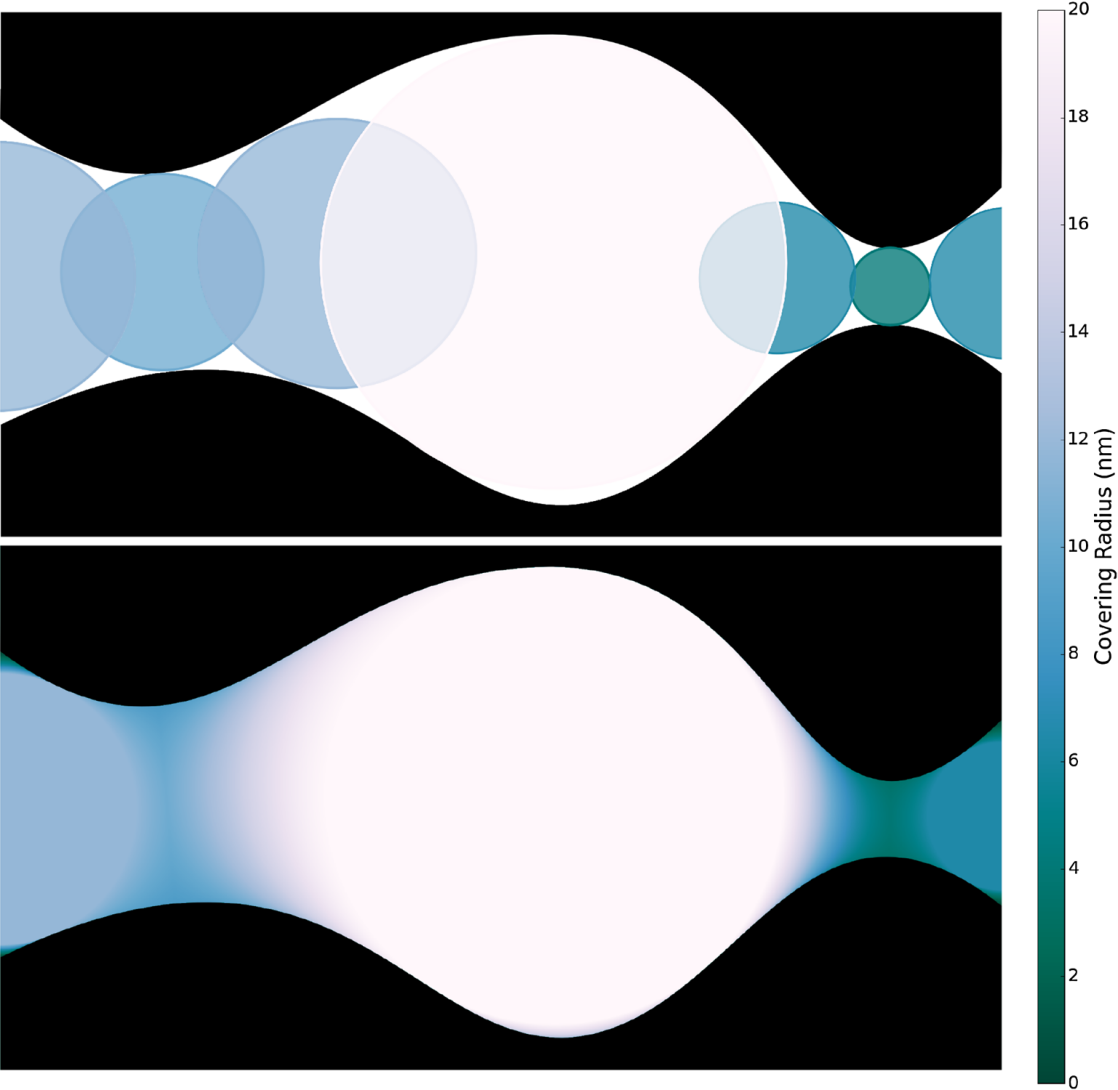
Covering radius transform example. In this two-dimensional example, an internal cavity is shown surrounded by two bottlenecks of different sizes. In the *top* figure are shown several *circular* probes, colored by radius. The *bottom* figure shows the covering radius transform, which indicates the largest such probe that could occupy each point in the void space

Given the EDT, one defines the CRT at a given point *x* ∊ *Ω* as the maximum radius of a hypothetical spherical particle contained in void space and overlapping *x*. These maximally inscribed spheres are described by the EDT. Thus the CRT, CRT : *Ω* *→* *R*, is defined in terms of the EDT as CRT(*x*) = max{EDT(*c*):*c* ∊ *Ω*, |*x* − *c*| ≤ EDT(*x*)}. Figure 4 shows the CRT computation for a hypothetical 2D pore. In the top panel of that figure, a collection of maximally inscribed spheres is shown, colored by radius. The maximum radius of all such maximally inscribed spheres corresponds to the CRT value at each point of void space, as shown in the bottom panel of Fig. 4.

Multiple algorithms exist for computing the CRT either directly using morphological methods [30] or using the EDT [26], as discussed in “Computation of the covering radius transform” section. Such methods exhibit poor asymptotic performance; however, the EDT-based method of Mickel et al. [26], which we use in this work, is quite parallelizable, making it suitable for use on large tomographic datasets.

The EDT and the associated CRT are useful for analyzing the sizes of spherical particles that could hypothetically fit in void space. However, considered as possible catalyst locations, many of these positions are infeasible because a large particle cannot traverse to that location from outside the cell wall due to obstruction by bottlenecks. Let Void(*r*) ⊂ *Ω* denote the subregion obtained by thresholding the distance function

$${\tt{Void}}\left( r \right) = \{ x \in \varOmega :{\tt{EDT}}\left( x \right) > r\} .$$

Whereas EDT(*x*) denotes the maximum size of a sphere which could be placed at position *x* without overlapping biomass, the thresholded set Void(*r*) denotes the collection of all points in the image domain at which a sphere of a given radius *r* could reside. Equivalently, Void(*r*) is the void space with a margin of size *r* around the biomass surface removed. Clearly the set Void(0) is simply the set of all void space, so that *Ω* = Biomass ∪ Void(0).

Each set Void(*r*) is composed of a number of connected components, which represent regions in which a particle could move continuously. Selecting connected components that overlap a given source region, such as the cell lumen, provides a collection of positions at which a sphere of radius *r* could not only reside, but could reach from the lumen without being blocked by bottlenecks. By decreasing the value of *r*, more positions become feasible so that the connected components of Void(*r*) grow and merge with one another. When two connected components merge in such a way, this represents a bottleneck connecting two regions. When the radius of the hypothetical sphere is small enough that it can pass through the bottleneck, a new region (connected component) becomes accessible, whereas spheres with radii larger than the bottleneck are separated into distinct connected components.

The covering radius describes the maximum size of a particle that could inhabit a particular location. However, as discussed, due to bottlenecks in a complicated matrix of biomass material found in the cell wall, large particles are often unable to actually reach internal pores and cavities. In order to quantify this effect, given a seed region *Σ* ⊂ *Ω* we define the *accessible Euclidean distance transform*, denoted aEDT_*Σ*_ : *Ω* *→* *R*, at a point *x* ∊ *Ω* as the maximum radius *r* > 0 such that there exists a continuous path from *Σ* to *x* contained entirely within Void(*r*):

$${\tt{aEDT}}_{\varSigma } \left( x \right) = { \hbox{max} }\{ r > 0:\exists \,{\text{continuous }}\gamma :\left[ {0,1} \right] \quad \to {\tt{Void}}\left( r \right),\gamma \left( 0 \right) \in \varSigma ,\gamma \left( 1 \right) = x\} .$$

At first glance, the aEDT seems formidable to compute, since it appears to require searching over all continuous paths within the image domain. However, as will be discussed in “Computing the aEDT using the contour tree” section, computation of the aEDT is made tractable by examining the structure of connected components of the sets Void(*r*).

The accessible covering radius, describes the largest spherical particle that could access the point *x* via diffusion from the seed region *Σ*. The *accessible covering radius transform*aCRT_*Σ*_ (*x*) is a function taking this value at each point *x*. Given the aEDT, the aCRT is computed in exactly the same way the EDT is used to compute the CRT. That is, at each point *x* of void space, each point within a sphere of radius aEDT_*Σ*_ (*x*) is visited. At each of those points *q* within the sphere, if the radius of the current sphere is larger than the current value of CRT_*Σ*_ (*q*), the aCRT is updated to be CRT_*Σ*_ (*q*) = aEDT_*Σ*_ (*x*).

#### Computing the aEDT using the contour tree

Connected component analysis of the thresholded EDT has been extensively studied in previous image processing and scientific visualization literature [31–34]. It is well-established that the contours and thresholded regions of the EDT form a hierarchical structure which is commonly represented by what is called the *contour tree*. Critical values of the EDT (local minima, local maxima, and saddle points) correspond to nodes of the contour tree, and contain rich information about the structure and relationship of connected regions found in each set Void(*r*). Thus, the contour tree provides a simplified representation of the segmented image, and as we will see it enables efficient analysis of accessibility for all possible particle sizes at once.

The contour tree contains leaves representing local extrema [32, 33]. In the case of the EDT, these leaves are local maxima. When these local maxima occur on the interior of the image domain, they represent pores or cavities: spaces that are only accessible by passing through bottlenecks. Local maxima are also possible along the image boundary, in which case they represent a possible source of particles from outside the imaged field of view.

In addition to leaves, the contour tree contains branches, which represent the joining of regions at bottlenecks. The root nodes of the tree represent the connected components of the biomass. The contour tree is often represented visually as a graph where each node is ordered vertically to represent its corresponding EDT value, as in Fig. 3. In this ordered representation, a horizontal line at a particular vertical position intersects the contour tree at points corresponding to the connected components of the contour. When varying the height of the horizontal line to cross a branch point in the tree, the number of intersected branches of the tree simultaneously changes, reflecting the merging of connected regions of padded void space at a bottleneck (e.g., the bottleneck labeled B in Fig. 3).

When computing the contour tree, we store a mapping from each voxel in the image volume to its associated edge in the contour tree. This allows us to work with the contour tree directly, tagging each point in the tree with a particular aEDT value as described below. The tagged contour tree is then used as a lookup table or index, enabling us to map function values back into the image volume after computing them on the contour tree.

**Fig. 5:**
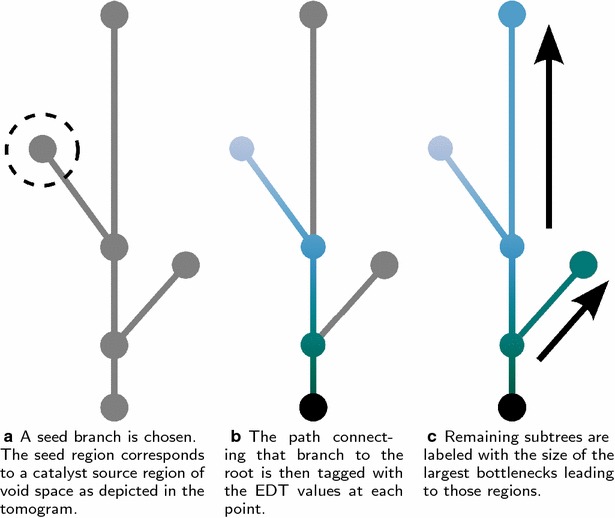
Computing accessibility on the contour tree. After choosing a seed branch **a** EDT values along the path from the seed to the root are preserved. **b** Then untagged subtrees are tagged according to the value at the lowest tagged branch point. **c** This value is the radius of the smallest bottleneck leading to the non-seeded subtree

In order to analyze accessibility, we introduce the accessibility-tagged contour tree. The contour tree, as described previously, has a natural “tagging” wherein each point in the tree is augmented with its corresponding EDT value during contour tree construction [32]. It is this tagging that is conventionally used to order the tree in the vertical representation, and it is also depicted in color in Fig. 3. Instead of tagging each point in the tree with the EDT value, the accessibility-tagged contour tree is tagged with the maximum radius for which that part of the tree is accessible. Tagging the contour tree with accessible radii is accomplished via a three-step process, as depicted in Fig. 5:

- First, a collection of seed leaves is selected. The seed leaves represent the seed region *Σ* in the image domain (described previously), which acts as a source of hypothetical diffusing spherical probes.
- In the second step, the tree is traversed downward from the seed leaves to the root nodes of the tree. As points in the tree are passed over, they are tagged with their corresponding EDT value. This models particle paths leaving the seed region through increasingly smaller bottlenecks, eventually reaching the biomass surface.
- Finally, in the third step, the tree is traversed upward from the root nodes. When an untagged subtree is encountered, the entire subtree is given a value corresponding to the earliest tagged branch encountered. Intuitively, this represents tagging the untagged region of the image volume with the size of the largest bottleneck leading to it from a more accessible region. This step accounts for all particle paths from the seed region that pass through small bottlenecks before reaching larger pores.

The accessibility-tagged contour tree is mapped back to the volume in a straightforward way. During contour tree construction, each voxel of the volume is tagged with a label indicating which segment of the contour tree it corresponds to. After computing the accessibility-tagged contour tree, these labels are used to look up the tag at each point of the tree. The aEDT is then the maximum of this tag and the EDT value at that voxel.

**Fig. 6:**
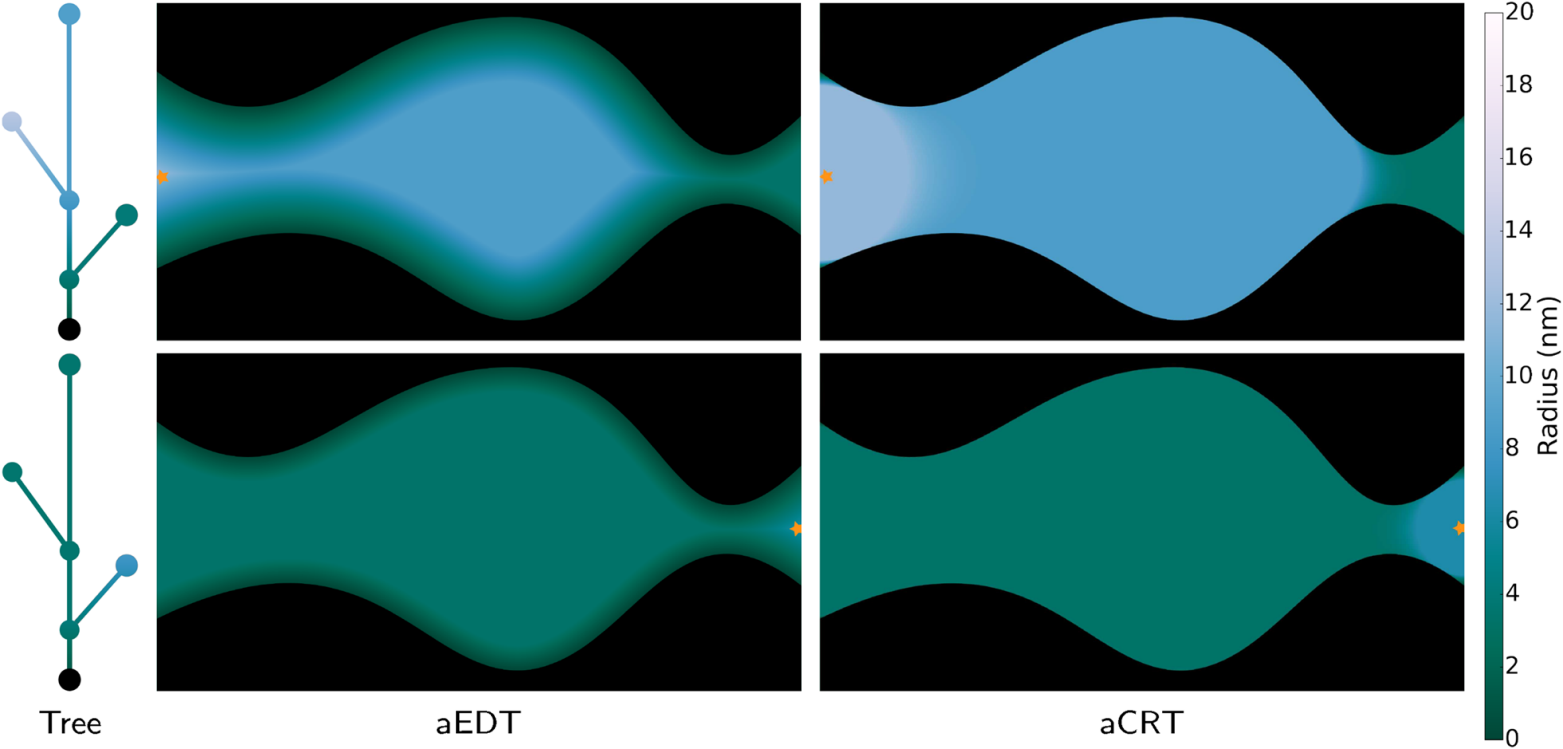
Shown here are accessibility-tagged contour trees (*left*), aEDTs (*center*), and aCRTs (*right*) for the cavity, seeded to show access from the left (*top*) and right (*bottom*) image boundaries. The accessibility-tagged contour tree is mapped back onto the volume in order to produce the aEDT. Whereas the EDT gives the maximum radius of a circle that could occupy each point without touching the biomass, an aEDT gives the maximum radius of a circle that could each each point by diffusing from a seed region. In each figure, the orange star indicates the seed region. The aCRT shows the maximum radius of a circle which could diffuse from the seed region and then overlap each point

From the aEDT, the accessible covering radius transform (aCRT) is computed in the same way the CRT is obtained from the EDT; by visiting each point of the aEDT, placing a sphere of the prescribed radius there and taking the maximum over all such spheres covering a given voxel. The result, as shown in Fig. 6 is a labeling of entire cavities with the radius of the bottlenecks leading into them.

### Surface accessibility

In the previous sections, we derived volumetric measures of accessibility, wherein at each voxel of the image domain the maximal radius of a diffusing particle was computed. However, the ultimate goal is to characterize the effect of accessibility on catalysis, a phenomenon that takes place when the catalyst interacts with the surface of the biomass. In this section, we describe a method for characterizing the portions of biomass surface that are accessible to the catalyst.

The aCRT describes the maximum accessible particle size that could come in contact with each point on the biomass surface by diffusion from the seed region *Σ*. For any given radius *r* > 0, any point *y* on the biomass surface Surf such that aCRT_*Σ*_ (*y*) ≥ *r* is a point that is accessible to spherical catalysts of radius *r* and smaller. Thus one measures the amount of surface accessible to a catalyst of size *r* by computing the surface area of {*y* ∊ Surf: aCRT_*Σ*_ (*y*) ≥ *r*}, the set of all surface points whose aCRT_*Σ*_ value is at least *r*. We call this surface area the accessible surface area associated with seed region *Σ* and radius *r*, and denote it by aArea_*Σ,r*_. Summarizing, the accessible surface area is computed as

$${\tt{aArea}}_{\varSigma ,r} = \mathop \int \limits_{\text{Surf}} \left[ {{\tt{aCRT}}_{\varSigma \left( y \right)} \ge r} \right]{\text{d}}y,$$

where the bracket [*P*] denotes the indicator function, which takes a value of one when its argument *P* is true and zero otherwise.

#### Surface accessibility and range of interaction

In the method presented above, one computes the surface accessibility by interpolating the aCRT along the surface of biomass. This is overly simplistic, however, since the aCRT is discontinuous at the biomass surface, taking values of zero inside the biomass, and possibly having very large values just across that boundary in the void space. In practice, this leads to nonsensical results if simple linear interpolation is used to compute values at the vertices of a triangular mesh from the voxel grid of the aCRT. However, note that this issue is due to the presence of nearby zero voxels, lying on the interior side of the biomass surface. The surface accessibility is meant to capture the largest size of a particle that could interact with the surface at each point, so in the continuum, we would naturally choose the value on the void space side of the boundary. In other words, one would choose the maximum of the nearby aCRT values when computing surface accessibility.

**Fig. 7:**
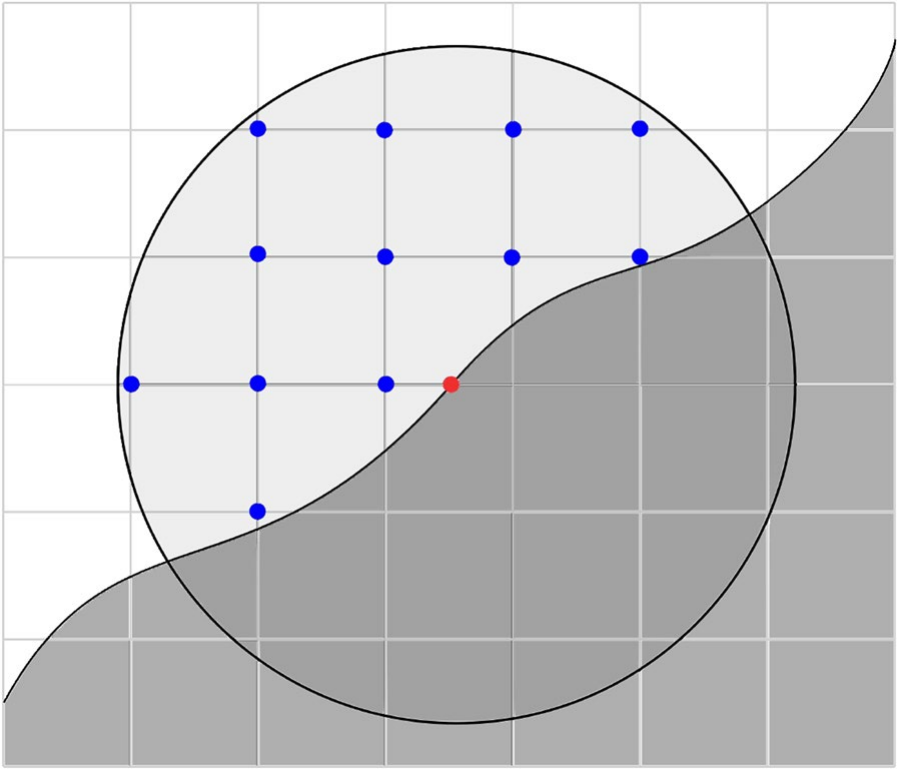
Surface accessibility and range of interaction. In this diagram, *white* denotes void space and *gray* denotes the internal biomass region. Points (*red*) on the biomass surface are assigned surface accessibility values by visiting all voxel locations (*blue*) within a sphere with radius equal to the specified range of interaction and computing the maximum aCRT value found at those voxels

We generalize this notion to include consideration of a small neighborhood of each point of the biomass surface. Let $\varepsilon > 0$ denote some small *range of interaction*, representing the maximum distance a catalyst needs to be from an object in order to chemically interact with it. As depicted in Fig. 7, at each point *y* ∈ Surf, the maximum radius of a catalyst that could interact with the point *y* up to this range is then computed by

$${\tt{aCRT}}_{\varSigma ,\varepsilon } (x) = \hbox{max} \{ {\tt{aCRT}}_{\varSigma } (z):\left| {z - y} \right| \leq \varepsilon \} .$$

In practice, we compute a triangular mesh, *m* ≈ Surf, approximating the biomass surface using the marching cubes algorithm [35]. When computing surface accessibility using tomography data, we always use a non-zero range of interaction to counteract partial volume effects. We have found empirically that ranges of interaction larger than about one voxel width are unnecessary for avoiding such adverse effects, and that larger ranges of interaction increase measured accessible surface areas without providing qualitative improvements to the accessibility surface maps.

#### Seed region strategies

The methods described above are able to characterize accessibility of biomass from any seed region within the tomogram volume. Of the utmost importance is accessibility from the lumen of cells, through which catalysts are easily transported. For some tomograms, such as those of native cell walls, the lumen is easily demarcated, and *Σ* can be obtained by simply flooding two regions of the tomogram on either side of the cell wall. When examining pretreated biomass, however, it is often necessary to manually segment the cell lumen. We perform lumen segmentation in the same step that we segment biomass, as is described in “Biomass segmentation” section.

While lumen accessibility is our focus, our ability to observe it is limited by tomogram thickness. This is because accessibility cannot be assessed in regions outside of the tomogram, whose image domain is typically rectangular and thin in a direction roughly axially aligned with a vascular cell. It is likely that there exist diffusion paths a catalyst might take from the lumen into the cell wall which pass outside the imaged volume. Such paths are not accounted for by our calculation of lumen accessibility alone, leading to a possible underestimation of accessibility. To accommodate this effect, in addition to seeding from the lumen, we also compute accessibility from the axial tomogram section boundary. This likely overestimates accessibility, since not every point on the image boundary is accessible from the lumen through a path outside the image domain. Essentially, this method only excludes cavities that are entirely resolved in the image domain, and otherwise coincides with the CRT-based accessibility measure. Along with accessibility from the segmented lumen, these two methods provide the best lower and upper bounds on catalyst accessibility that can be expected from a finite thickness tomogram.

### Pretreated biomass accessibility

Corn stover samples, pretreated by three different pretreatments as well as unpretreated (native), were imaged according to the process described in “Sample preparation and tomographic imaging” section. For each dataset, a tomogram was constructed and segmented as described in “Biomass segmentation” section. A slice of each tomogram is shown in Fig. 1 and the biomass segmentation is shown in Fig. 2. The volumes and surface areas of segmented biomass are presented in Table 1. All of the reconstructed tomograms were of similar size, with similar-sized isotropic voxels. Notice that the more heavily pretreated samples (DA/SE and AFEX) have much higher surface area to volume ratios.

**Table 1:** Volumes and surface areas

	Tomo. sz. (nm)	Voxel sz. (nm)	Bio. vol. (µm^3^)	Area (µm^2^)	Area/vol. (µm^−1^)
Native	1580 × 1960 × 136	2.1	0.309	0.663	2.14
DA/ZC	1980 × 2050 × 107	2.2	0.346	1.96	5.66
DA/SE	1820 × 1950 × 116	2.2	0.227	17.0	74.7
AFEX	1870 × 1870 × 126	2.0	0.194	25.3	130

Shown here are the sizes of each tomogram. In addition, the volume and surface area of segmented biomass are listed along with surface area to volume ratio

**Fig. 8:**
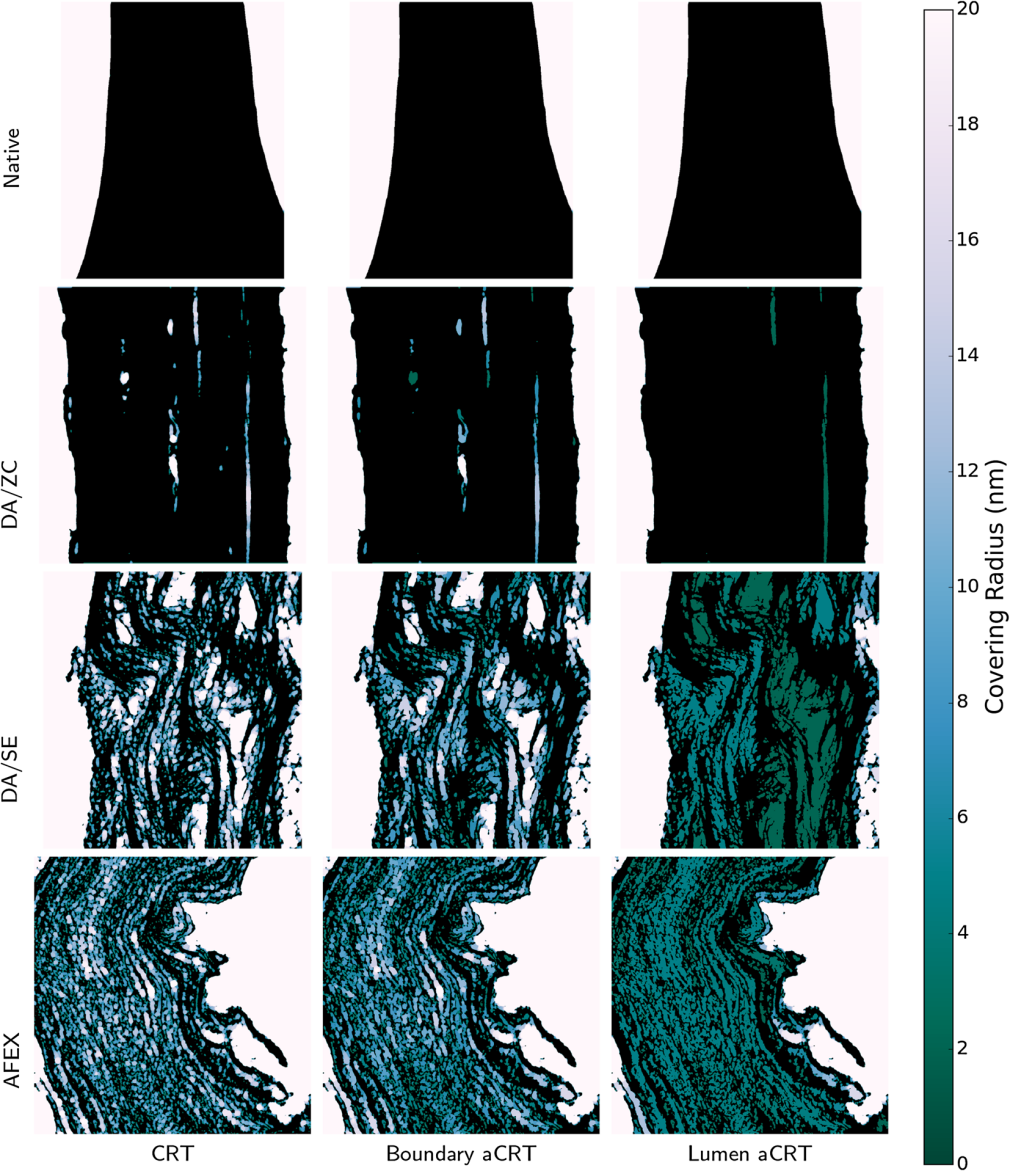
Accessible covering radius transform. *Color* indicates the accessible covering radius transform value, with *lighter colors* indicating higher covering radii. Each *row* shows one of the four datasets, while each *column* shows a different method of seeding the aCRT, corresponding to a different notion of accessibility. Notice that nearly all internal cavities in the DA/ZC dataset are completely inaccessible from the lumen, and the internal regions of both the DA/SE and AFEX datasets are considerably less accessible to the lumen than they are from the boundary

The CRT, boundary aCRT, and lumen aCRT were computed for each dataset and the results are shown in Fig. 8. The internal delaminations present in the DA/ZC dataset are shown as less accessible using the boundary method, and are shown as nearly completely inaccessible when using the lumen-based notion of accessibility. Notice that boundary accessible covering radius values lie somewhere between CRT-based and lumen-based accessible covering radius values.

**Fig. 9:**
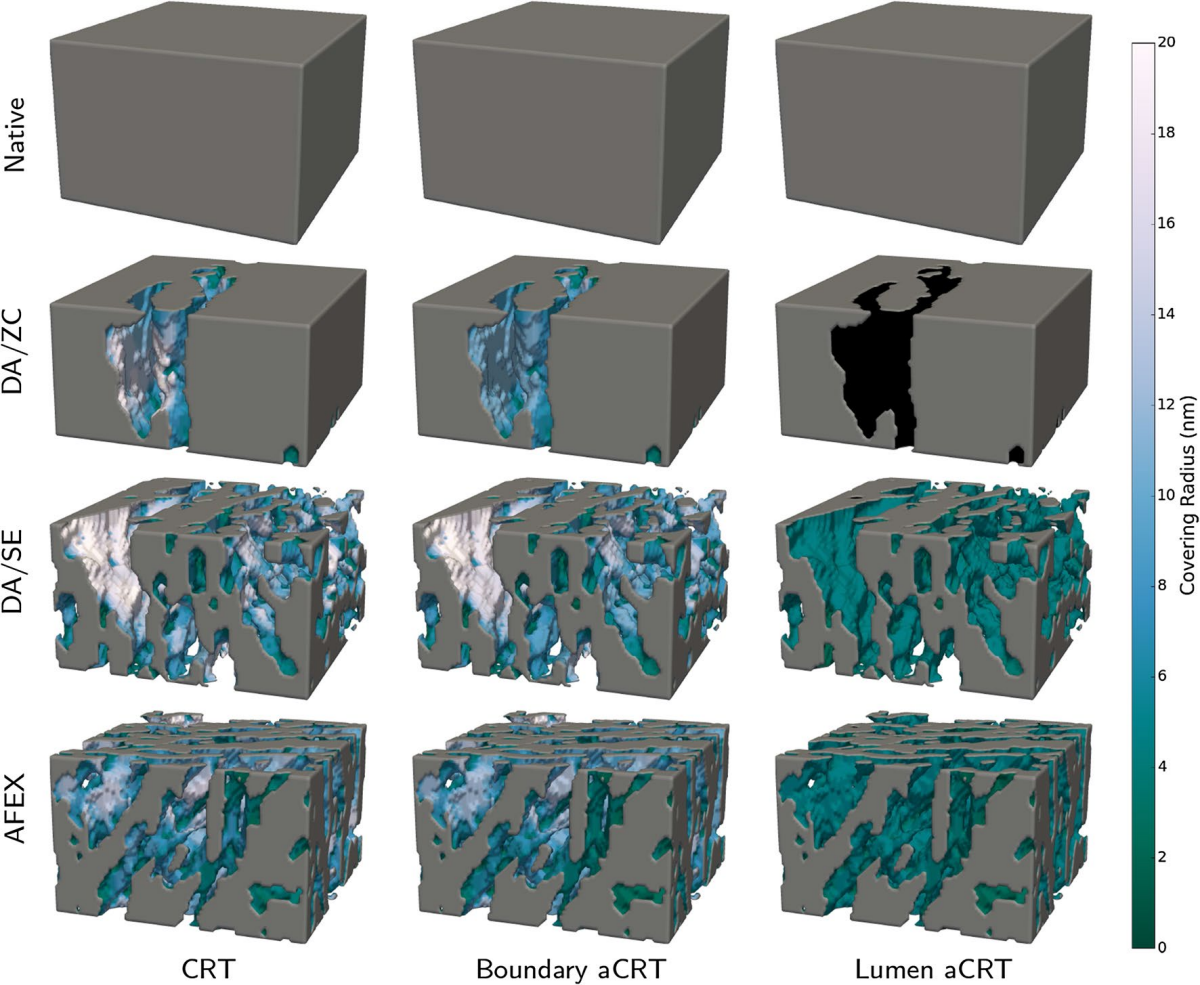
Surface accessibility functions. After computing a surface using a biomass segmentation, the aCRT is interpolated to compute, for each point on the biomass surface, the maximum radius of a spherical probe that could contact the surface at that point. A 2.2-nm range of interaction was used for all of the computations. Shown here is a representative region of each dataset, with coloring given by the surface accessibility value. The subregions shown correspond to the interior regions indicated in Fig. 2. *Gray* surfaces denote internal biomass regions and do not represent actual biomass surface

In order to compute surface accessibility maps, the biomass surface was generated using marching cubes [35]. Surface accessibility maps were then computed using an interaction distance of 2.2 nm for all datasets, which is just over one voxel width in each case. Representative small subregions of all of the surface accessibility maps are shown in Fig. 9. The native dataset shows identical surface accessibility using each method. This is expected, since the native dataset contains no internal cavities or bottlenecks, so that its only biomass surfaces are the lumen surfaces. For each of the other datasets, at least one internal cavity is shown with a higher value in the CRT-based surface accessibility map than in the boundary-based accessibility map, which shows that internal cavities that are fully resolved are affected by restricting accessibility to the tomogram boundary. Clearly, lumen-based accessibility is the most extreme measure, as entire regions of the sample regions are closed off to all but the smallest catalysts.

**Fig. 10:**
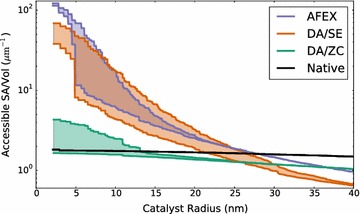
Surface accessibility versus catalyst radius. Shown above is a semilog plot of accessibility versus probe radius for each of four datasets. Using surface accessibility maps, the area of biomass surface accessible to probes of at most a given radius are computed. In order to fairly compare datasets, accessible surface area is divided by the total volume of biomass in the segmented tomogram. Lower bound curves are obtained by seeding from the cell lumen, while upper bounds are obtained by seeding from the axial tomogram boundary. The resulting plots show that as probe radius increases, less biomass surface can be contacted. Clearly, the AFEX dataset shows the most accessibility to catalyst radii below 5 nm. However, for catalyst radii in the range of 8–20 nm, AFEX and DA/SE exhibit similar surface accessibility. The native and DA/ZC datasets have much less biomass surface overall as shown in Table 1, which is expected as AFEX and DA/SE appear to have more thoroughly deconstructed the cell wall. For catalysts above 20 nm radius it appears the accessible surface to volume ratio is not particularly increased by even the AFEX pretreatment. In fact, due to increased luminal surface irregularity in the pretreated samples (see Fig. 2,* bottom row*), the surface area to volume ratio at such high catalyst radii is decreased relative to native

Shown in Fig. 10 is a semilog plot of accessible surface areas as a percentage of the total biomass surface area, computed for each dataset. As justified in “Seed region strategies” section, lower bounds are computed using the lumen seeding strategy as described above, while seeding from the section boundary provides upper bounds. Because the native dataset has no internal cavities or channels, both methods result in the same curve. In that case, the gradual decrease in accessible surface area is due to the texture of the boundary of the cell wall, with smaller probes able to reach indentations but never reaching any bottlenecks.

On the other hand, each of the other three datasets shows dramatic decreases in accessible surface area as catalyst radius is increased. For the DA/ZC dataset, which includes long delaminations that are not accessible from the lumen, the lumen-based accessibility curve is relatively flat, resembling that of the native dataset. This is because those internal laminations are not accessible to the lumen, so that in the lumen-based accessibility lower bound, the DA/ZC dataset is very similar to a native dataset, having only the lumen-cell wall boundary accessible to catalyst.

The DA/SE and AFEX datasets each show considerably more deconstruction and nanofibrillation than the DA/ZC dataset. For these datasets, even in the lumen-based accessibility lower bounds in Fig. 10 there is significantly more biomass access for catalysts below a threshold of roughly 5–10 nm radius. Above those sizes, the deconstructed cell wall contains enough obstacles to prevent penetration of the cell wall to any significant depth, as is also seen in Fig. 8. This suggests that catalysts below around 5 nm radius have significantly more access to biomass in both the DA/SE and AFEX datasets than do larger catalysts.

In Fig. 10, it is readily seen that at high catalyst radii (above about 28 nm), each of the pretreated samples exhibit slightly lower surface accessibility than native. This is likely due to increased irregularity in the lumenal surface of the cell wall following pretreatment. This increased roughness causes large catalysts to only contact the lumenal surface at small ridges, decreasing the amount of accessible surface area relative to that of samples with smoother lumenal surfaces. This lumenal surface roughness is seen in the bottom row Fig. 2, with increasing roughness corresponding to sharper decreases in accessibility at large radii in Fig. 10.

## Conclusion

Our results indicate that image-based surface accessibility provides a useful quantification of accessibility of biomass to catalyst. In addition, visualization of the aCRT provides useful qualitative information about the spatial heterogeneity of accessibility across the cell wall. In Fig. 8, the lumen aCRT figure for the DA/SE sample shows uniform coloration within distinct inter-lamellar regions, indicating that lamella are a microstructural feature with large influence on catalyst accessibility. Delamination is the dominant mechanism for increased accessibility when no nanofibrillation is observed, as is clearly observed in our DA/ZC dataset.

The accessible surface area measurements we have presented give a useful perspective on just how much pretreatments such as DA/SE and AFEX increase accessibility. The tomography data (Fig. 1) show that the biomass is drastically altered by such methods and a simple surface area to volume ratio calculation (Table 1) shows an increase of 1–2 orders of magnitude in that ratio. However, as Fig. 10 shows succinctly, this extreme increase in accessibility is only available to very small catalysts.

At large catalyst radius, our method exposes a decrease in surface accessibility for pretreated biomass due to increased lumenal surface roughness. This surface roughness contributes to an increase in the overall surface area to volume ratio, but using our measure we see that, as with the increased surface area due to delamination, only small catalysts are able to take advantage of that increase.

The results put forth in this paper are meant to be interpreted in the context of current catalysis work. Enhancing biomass accessibility to catalysts remains a major challenge to improving cellulose digestibility. Our analysis shows that using existing pretreatment methods, biomass accessibility is increased dramatically for small inorganic catalysts. However, we observe that this is not the case for larger multi-unit enzymes, which are unable to pass through bottlenecks near to lumenal surfaces even in harshly pretreated biomass.

Single-unit enzymes such as Cel7A have radii in the vicinity of 5–6 nm [6], and as seen in Fig. 10 may be able to benefit somewhat from the increase in accessible surface area from steam explosion pretreatments. However, larger enzymes having multiple subunits connected by cellulose-binding-modules (CBM), such as CelA, have radii around 7–15 nm [9]. We have shown that enzymes of that size cannot take full advantage of increased surface area directly, and that the area of biomass surface accessible to them is similar to that of native. Indeed, it has been observed that CelA is capable of a cavity-forming process of its own during digestion, which may be responsible for its enhanced digestion performance despite the lack of biomass accessibility due to its size [9]. Similarly, we have shown that the increases in surface area due to nanofibrillation and delamination are on a scale that is insufficient to explain the increased performance of even larger multi-enzyme complexes such as the cellulosome *C. thermocellum*, which have radii in the 50–70 nm range [10].

Tomography-based analysis methods, such as the one presented in this work, are limited by the resolution and field of view available in single tomogram. However, state-of-the-art tomography methods are able to resolve individual cellulose microfibrils in deconstructed biomass and, as is clear from our results, the entire width of cell walls can be imaged at once. It is natural to attempt to increase the field of view in the axial direction through serial sectioning and serial tomography techniques, and we intend to apply these techniques in the future. However, it is important to note that such serial techniques unavoidably introduce gaps between sections, which must be handled delicately. Though we expect serial studies to reveal important information about the variability of our accessibility measures, we also point out that we expect that serial methods will not increase the boundary accessibility upper bound used in the present work. Rather, we expect that future studies using serial imaging methods will primarily serve to shrink the gap between our boundary-based and lumen-based accessibility measures and provide more precise characterization of true catalyst accessibility.

## Methods

### Sample preparation and tomographic imaging

#### Biomass pretreatment

Chemical and physical substrate analysis of the DA/SE pretreated corn stover was reported previously [36]. DA/SE pretreatment was carried out using methods previously reported [37, 38]. Briefly, corn stover (Pioneer variety 33A14) from the Kramer farm in Wray, Colorado was tub ground, then milled through a Mitts and Merrill rotary knife mill (model 10 × 12) to pass a 1/4-in. screen. Acid impregnation was carried out using 120 L of ~45 °C 0.5 wt% H_2_SO_4_ in a 200 L recirculation tank. For dilute acid/Fe^3+^ ion co-catalyst impregnation, the acid was equilibrated for 4 h in the recirculating bath. A Hastelloy C-276 wire 20 mesh basket was loaded with 14.5 kg of 1/4-in. milled corn stover feedstock (~94 % solids) and lowered into the bath of warm dilute acid/Fe^3+^ ion co-catalyst for 2 h. The biomass was drained of excess acid to approximately 20 % solids and loaded into a hydraulic dewatering press where the acid impregnated feedstock was pressed to ~45 % solids. A 4 L Hastelloy steam explosion reactor was prewarmed to pretreatment temperature and was loaded with 500.0 g of dilute acid/Fe^3+^ ion co-catalyst impregnated and pressed feedstock (~45 % solids) and quickly heated (~5–10 s) via direct steam injection to 150 °C. At 15 min, the pretreated feedstock was rapidly depressurized to atmospheric pressure and blown into a flash tank.

Zipper-clave dilute acid pretreatment was carried out in a 4 L ZC^**®**^ vertically stirred reactor (Autoclave Engineers, Erie, PA, USA). Pressed dilute acid impregnated feedstock (160 g) was inserted into the reactor and steam was injected directly into the bottom through ports in the agitator and constant temperature was imposed by controlling the steam pressure in the reactor. The contents within the ZC reactor typically reached the target temperature within 5–10 s of the onset of steam flow. Following the reaction period, the steam pressure was slowly released through a condenser over 15–30 s to lessen boil-over and the pretreated solids were sealed in a plastic freezer tub and stored at 4 °C for later analysis.

Chemical and physical substrate analysis of the AFEX-pretreated corn stover was reported previously [36]. AFEX pretreatment was carried out as described previously [39]. Briefly, pretreatment on NREL corn stover was conducted in a 2-L Parr reactor (316 SS, PARR Instrument Co., Moline, IL). The reactor was clamped shut and 1:1 ammonia to biomass loading was injected using a preweighed ammonia delivery vessel. The reactor was heated using a custom aluminum block on a hot plate and maintained at 130 °C for 15 min. At the end of the residence time, the pressure was explosively released. The biomass was removed from the reactor and left overnight to remove the residual ammonia.

#### Sample preparation for TEM and image acquisition

Pretreated biomass samples were dehydrated by treating with increasing concentrations of acetone by increments of ~20 vol % and intermittently heated for 1 min in a Pelco microwave oven after each addition of acetone. After dehydration, the samples were infiltrated with Eponate 812 (EMS, Hatfield, PA) by incubating at room temperature for several hours to overnight in increasing concentrations of resin in increments ~20 vol % diluted in acetone until 100 % resin was reached, after which three complete resin exchanges were performed. The infiltrated samples were transferred to capsules and the resin polymerized in and oven at 60 °C overnight. Samples embedded in resin blocks were sectioned to ~250 nm with a Diatome diamond knife on a Leica EM UTC ultramicrotome (Leica, Wetzlar, Germany). Sections were collected on 0.5 % Formvar coated slot grids (SPI Supplies, West Chester, PA) and were post-stained for 2 min with 1 % aqueous KMnO4. Images were captured with a four mega-pixel Gatan UltraScan 1000 camera (Gatan, Pleasanton, CA) on a FEI Tecnai G2 20 Twin 200 kV LaB6 TEM (FEI, Hillsboro, OR) using SerialEM [40].

#### Image acquisition

Over a hundred micrographs of prepared samples were individually analyzed. Using these micrographs, the 50 best-prepared samples were selected and used to obtain tomograms. Tomograms were obtained by first capturing dual-axis ±60° tilt series of 2 × 2 montage frames of the regions of interest at a pixel size of ~0.5 nm. Tomographic reconstructions were constructed from the tilt series using the R-weighted back projection algorithm within the IMOD software package [41]. Single-axis tomograms were then combined to yield dual-axis tomograms using a warping algorithm [40, 42]. The high-resolution tomograms were then downsampled. It was manually verified that the downsampling had little effect on the observable structures found in each tomogram. Exact tomogram sizes after this preprocessing are given in Table 1. Of the 50 analyzed tomograms, those four with the least image artifacts were used for detailed accessibility analysis in this study.

### Biomass segmentation

Tomograms were used to obtain volumetric segmentations of biomass. The contrast in our samples was generally sufficient to easily identify biomass. Note that we are currently unable to reliably distinguish between cellulose, hemicellulose, lignin, and other constituent materials of plant cell walls. Because of that, we label all dark parts of the tomogram in the area of the cell wall as “biomass.”

**Fig. 11:**
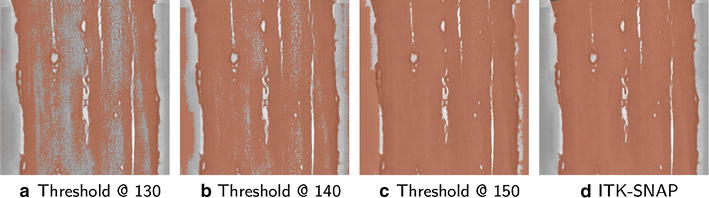
Manual versus semi-automatic segmentation. Shown above is the DA/ZC dataset (*grayscale*) overlaid with segmentations (*orange*) resulting from thresholding at intensity values 130, 140, and 150 (arb. units), as well as the result of semi-automatic region-growing segmentation using the ITK-SNAP software [43]. Thresholding at too low an intensity value (**a**) under-segments the interior cell wall, leaving much of the biomass in the cell wall mislabeled as void space. As the threshold intensity is increased (**b**), this under-segmentation problem is reduced. However, in order to eliminate under-segmentation of the interior of the cell wall, one must choose a threshold value so high that the lumen is over-segmented (**c**). Through user intervention using the convenient ITK-SNAP tool, a segmentation (**d**) is produced in which biomass in the interior of the cell wall is correctly identified, while avoiding mislabelling dark regions in the lumen as biomass

A common and simple method of segmentation is global thresholding, wherein voxels whose tomogram value fall below a specified cutoff are labeled as biomass. This method is useful as a first attempt at segmentation, but often more sophisticated methods are needed. Particularly in tomography data, the contrast often varies spatially, necessitating different cutoff values for thresholding in various regions of the image [24]. This problem is illustrated in Fig. 11, wherein it is shown that for the DA/ZC dataset, a threshold level cannot be chosen such that it provides a satisfactory segmentation at all points in the tomogram. We use a semi-automatic segmentation method based on active contour region-growing, implemented in the open-source ITK-SNAP software ([43], http://itksnap.org). Though other semi-automatic segmentation tools such as Amira (http://amira.com) and Seg3D (http://seg3d.org) would likely provide similar segmentation results, we chose ITK-SNAP because of its ease of use and ability to handle large datasets. Volumes and surface areas of the segmented biomass regions are given in Table 1.

### Computation of the covering radius transform

Given an EDT or aEDT function *D*(*x*), defined on an isotropic voxel grid, we compute the covering radius transform as follows. First we initialize the function CRT(*y*) to zero at every voxel. At each voxel *x*, we visit every voxel *y* such that |*y* − *x*| ≤ *D*(*x*). At every such *y*, we set CRT(*y*) to max[CRT(*y*)*, D*(*x*)]. This operation is rather computationally demanding, having complexity ${\mathcal{O}}(mn)$, where *n* is the number of voxels of void space and *m* is the average number of voxels in the spheres of *D*(*x*). However, a speedup is available via parallelization over the output voxels. Additionally, as discussed by Hildebrand and Rüegsegger [27], another massive speedup is obtained by considering so-called non-redundant spheres, whose centers lie along the ridges of *D*(*x*).

## Abbreviations

EDT: Euclidean distance transform
CRT: covering radius transform
aEDT: accessible Euclidean distance transform
aCRT: accessible covering radius transform
aArea: accessible surface area
DA/ZC: dilute acid plus zipper-clave pretreatment
DA/SE: dilute acid plus steam explosion pretreatment
